# RNA Binding Proteins that Control Human Papillomavirus Gene Expression

**DOI:** 10.3390/biom5020758

**Published:** 2015-05-05

**Authors:** Naoko Kajitani, Stefan Schwartz

**Affiliations:** Department of Laboratory Medicine, Lund University, Lund 22184, Sweden; E-Mail: Naoko.Kajitani@med.lu.se

**Keywords:** HPV, papillomavirus, splicing, polyadenylation, SR proteins, hnRNP, SRSF1, SRSF9, hnRNP A1, hnRNP D

## Abstract

The human papillomavirus (HPV) life cycle is strictly linked to the differentiation program of the infected mucosal epithelial cell. In the basal and lower levels of the epithelium, early genes coding for pro-mitotic proteins and viral replication factors are expressed, while terminal cell differentiation is required for activation of late gene expression and production of viral particles at the very top of the epithelium. Such productive infections are normally cleared within 18–24 months. In rare cases, the HPV infection is stuck in the early stage of the infection. Such infections may give rise to cervical lesions that can progress to cancer, primarily cancer of the uterine cervix. Since cancer progression is strictly linked to HPV gene expression, it is of interest to understand how HPV gene expression is regulated. *Cis*-acting HPV RNA elements and cellular RNA-binding proteins control HPV mRNA splicing and polyadenylation. These interactions are believed to play a particularly important role in the switch from early to late gene expression, thereby contributing to the pathogenesis of HPV. Indeed, it has been shown that the levels of various RNA binding proteins change in response to differentiation and in response to HPV induced cervical lesions and cancer. Here we have compiled published data on RNA binding proteins involved in the regulation of HPV gene expression.

## 1. Introduction

Human papillomaviruses (HPV) are small DNA viruses with a strict tropism for human epithelial cells [1,2]. They may be divided into cutaneous and mucosal types depending on the type of epithelium they infect. The mucosal types are mainly sexually transmitted. In general, HPV infections are asymptomatic and persist for 18–24 months before they are cleared by the immune system of the host. Cutaneous HPV types such as HPV1 may cause skin warts that normally regress spontaneously, whereas the genital, sexually transmitted HPV types such as HPV6 and HPV16 may cause either genital warts (condylomas), or remain asymptomatic until they are cleared. In rare cases, sexually transmitted HPVs of high-risk type such as HPV16 may establish chronic persistent infections that remain in their host for years or decades [3]. These infections may cause cervical high-grade lesions that constitute a risk factor for development of cervical cancer. More than 99% of the cervical cancers contain HPV DNA [4]. HPV is associated with other anogenital cancers as well, but less strongly than with cervical cancer, e.g., anal cancer, vulvar cancer and penile cancer. It is also well established that HPV is associated with oropharyngeal carcinomas. Epidemiological studies have established that the most common high-risk HPV type is HPV16, and that HPV16 is present in approximately 50% of the >500,000 cases of cervical cancer diagnosed annually worldwide [5,6]. At the molecular level, it is of great interest to investigate how HPV16 can transform the infected cell to a cancer cell and how HPV16 can establish persistence without being detected by the immune system of the host. The latter is amongst all attributed to the strict control of HPV gene expression by viral and cellular factors.

## 2. HPV Molecular Biology

**Figure 1:**
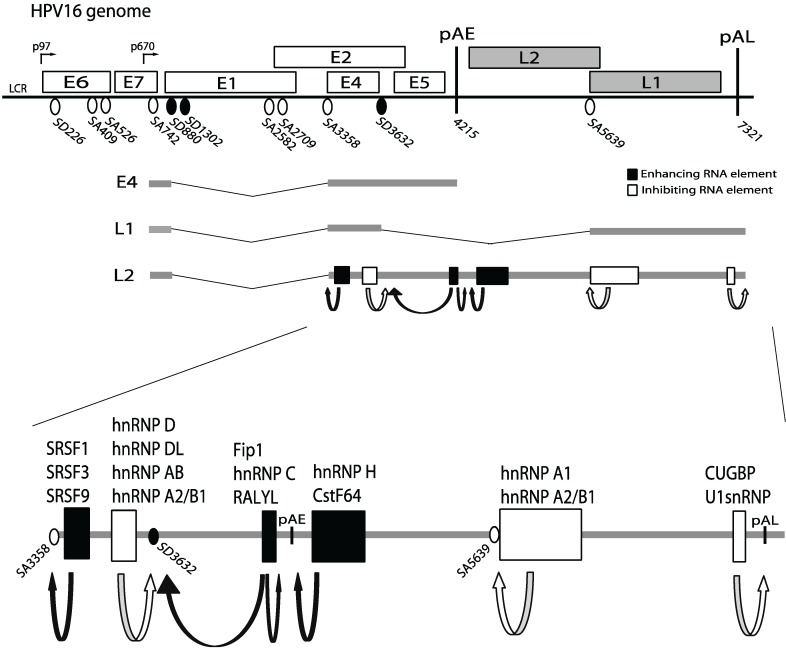
Schematic representation of the HPV16 genome. Boxes indicate open reading frames. HPV16 early and late promoters p97 and p670, respectively, early and late polyadenylation signals pAE and pAL, respectively, and 5'- (SD) and 3'- (SA) splice sites are indicated. A subset of HPV16 mRNAs initiating at the p670 promoter is shown and splicing regulatory elements are indicated. Filled boxes represent positive RNA elements that enhance usage of splice sites or polyA signals and white boxes represent suppressive RNA elements that inhibit splice sites or polyA sites. Examples of alternatively spliced HPV16 mRNAs initiated at early (p97) and late (p670) promoters and terminated at early (pAE) or late (pAL) polyadenylation signals are displayed.

The HPV virion is approximately 50 nm in diameter and consists of eight kilobases, double stranded, circular DNA genome embedded in an icosahedral capsid formed by the HPV L1 and L2 capsid proteins [7]. The viral genome contains a non-coding region (NCR) located between the end of the late L1 coding region and the beginning of the early E6 coding region (Figure 1). It encodes an origin of DNA replication, binding sites for the HPV E2 replication- and transcription-factor and the HPV early promoter. In HPV16, the early promoter is termed p97 and could potentially be used to express viral mRNAs encoding all HPV proteins. However, HPV mRNAs are either polyadenylated at the early polyadenylation signal (pAE), located downstream of the early genes (E1, E2, E4–E7), or at the late polyadenylation signal (pAL) located downstream of the late genes L1 and L2 [8,9]. At the early stage in the viral life cycle, mRNAs are transcribed from the early promoter p97 and polyadenylated at pAE, while at the late stage the late promoter p670 located in the E7 coding region is activated (Figure 1) [7,10,11]. Initiation at p670 excludes transcription of the E6 and E7 genes, but produces “early” mRNAs encoding E1, E2, E4, E5 that are polyadenylated at pAE, and late mRNAs encoding L1 and L2 that are polyadenylated at the late polyadenylation signal pAL. The relative levels of the individual HPV mRNAs are determined by alternative splicing and polyadenylation (Figure 1) [9,12,13]. Therefore, there is a switch from the early stage of the viral life cycle in which all early genes are expressed, to the late stage in which all early genes but E6 and E7 are expressed as well as the two late structural proteins L1 and L2. The switch from early to late HPV gene expression is dependent on cell differentiation. HPV L1 and L2 late gene expression is required for virus production and normally occurs in terminally differentiated cells at the very top of the epithelium.

## 3. HPV Gene Regulation

HPV gene expression is regulated at the level of RNA synthesis [14,15,16] and RNA processing [9,12,17]. Regulation of transcription involves a promoter switch from the early to the late promoter, which causes a down-regulation of expression of the pro-mitotic E6 and E7 proteins, and activation of the late L1 and L2 genes in a cell-differentiation dependent manner [7,10,11]. The late promoter also drives expression of E1, E2 and E4 proteins while the expression of E5 is more enigmatic. Entry into the late phase of the HPV life cycle initially results in a burst of HPV DNA replication as a result of the high E1- and E2-expression, which is followed by high E4 production and subsequent activation of L1 and L2 expression. While a promoter switch is necessary for entry into the late stage of the viral life cycle, a read-through at the early HPV polyadenylation signal pAE and activation of the late polyadenylation signal pAL are also required. Regulation of HPV alternative polyadenylation is therefore important in the HPV replication cycle. However, the promoter- and polyadenylation-switch are not sufficient to induce HPV late gene expression. A change in HPV alternative splicing and activation of HPV late splice sites is equally important. Regulation of HPV alternative polyadenylation and splicing is executed primarily by cellular RNA-binding proteins [12,13,17], although HPV E2 appears to affect HPV RNA splicing *in vitro* [18]. Recently the HPV E2 protein was shown to control HPV early polyadenylation thereby having the potential to act as an inducer of HPV late gene expression [19]. These results suggest that HPV E2 plays an active role in the control of HPV RNA processing [12].

## 4. *Cis-*Acting RNA Elements that Control HPV Gene Expression

Expression of the HPV genes is regulated at the level of transcription and the switch from the early to the late gene expression program involves a promoter switch from the HPV16 early p97 promoter to the late, differentiation-dependent promoter p670 (Figure 1) [7,10,11]. Since both promoters are located relatively close to each other at the 5'-end of the genome, regulation of HPV16 gene expression and in particular the switch from early to late gene expression occurs at the level of RNA processing, including alternative splicing and polyadenylation (Figure 1) [9,12,13,17]. A number of splicing and polyadenylation regulatory elements that interact with cellular proteins have been identified in HPV16 (Figure 1) [9,12,13,17]. Interestingly, sequence polymorphism within HPV16 may affect binding of cellular proteins to HPV16 mRNAs [20]. Strong splicing enhancer elements have been mapped to sequences downstream of the HPV16 3'-splice site SA3358, the most commonly used 3'-splice site on the HPV16 genome [21,22,23]. The splicing enhancer appears to be composed of an AG-rich element that is followed by an AC-rich element, both interacting with SR-proteins (SRSF1, SRSF3, and SRSF9) (Figure 1) [13,22,23,24,25]. Early mRNAs spliced to SA3358 are polyadenylated at pAE to generate E6 and E7 producing mRNAs. The early polyA signal is efficiently used at the early stage of the HPV life cycle to prevent read-through into the late region. Upstream, U-rich sequences in the early UTR bind to Fip1 and promote polyadenylation at pAE [26]. In addition to those, sequences located downstream of pAE in the L2 coding region interact with hnRNP H and CstF-64 to enhance polyadenylation at pAE [27,28,29,30]. During the early stage of the infection, HPV16 late gene expression is efficiently suppressed by inhibitory RNA sequences. Inhibitory HPV16 RNA elements suppress the two HPV16 late splice sites SD3632 and SA5639 that are used exclusively by HPV16 late mRNAs [9,12]. Late 5'-splice site SD3632 that is located between 3'-splice site SA3358 and pAE, and is used in a mutually exclusive manner to pAE, is strongly suppressed by slicing silencer RNA elements immediately upstream of S3632 [21,31]. This splicing silencer encodes two AUAGUA motifs that bind proteins of the hnRNP D-family and hnRNP A2/B1 to suppress SD3632 [31]. Late 3'-splice site SA5639 is also suppressed by splicing silencers. These RNA elements consist of AU-rich sequences that interact with hnRNP A1/hnRNP A2/B1 and are located in the L1 coding region downstream of SA5639 [32,33,34,35]. In addition to these splicing inhibitory elements, the late polyA signal pAL is strongly suppressed by negative RNA elements located in the late UTR that interact with U1snRNP and CUGBP [36,37,38,39,40]. Thus, HPV16 gene expression is controlled by a number of *cis*-acting RNA elements that either enhance or suppress splice sites or polyA signals (Figure 1). Previous reviews have addressed RNA regulatory elements in bovine papillomaviruses and human papillomaviruses [9,12,13,41] or specific aspects of papillomavirus RNA processing such as elements in the untranslated regions of papillomavirus mRNAs [17] or structures of alternatively spliced papillomavirus mRNAs [8,9]. In this review, we have compiled results on RNA binding proteins involved in human papillomavirus gene regulation.

## 5. Cellular RNA Binding Proteins that Control HPV Gene Expression

### 5.1. hnRNP A1

**Figure 2:**
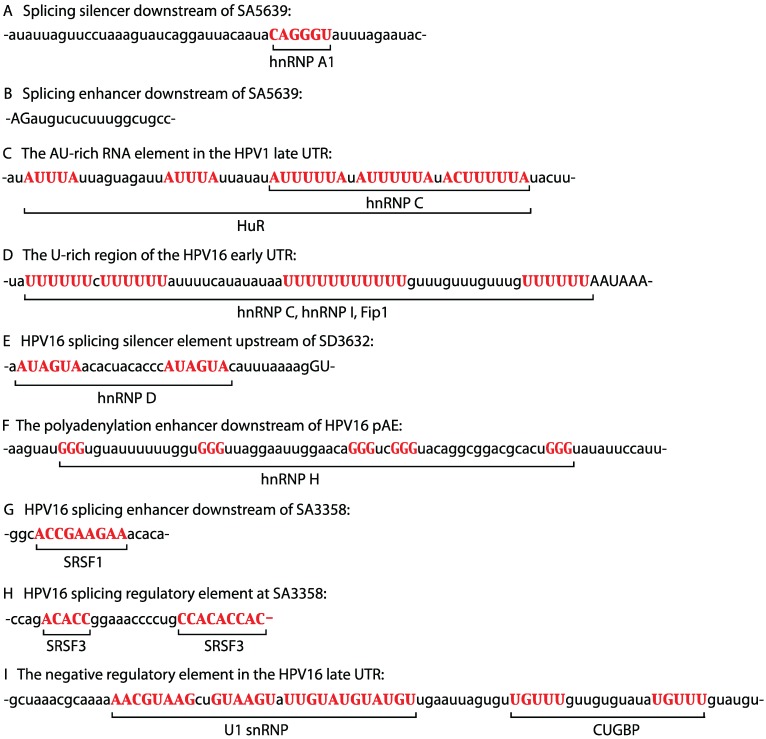
Sequences of *cis*-acting RNA elements on the HPV1 or HPV16 genome. Binding sites for cellular factors are capitalized and in red. See text for details. (**A**) Splicing silencer downstream of SA5639. (**B**) Splicing enhancer downstream of SA5639. (**C**) The AU-rich RNA element in the HPV1 late UTR. (**D**) The U-rich region of the HPV16 early UTR. (**E**) HPV16 splicing silencer element upstream of SD3632. (**F**) The polyadenylation enhancer downstream of HPV16 pAE. (**G**) HPV16 splicing enhancer downstream of SA3358. (**H**) HPV16 splicing regulatory element at SA3358. (**I**) The negative regulatory element in the HPV16 late UTR.

hnRNP A1 has been shown to bind specifically to splicing silencer RNA elements in the HPV16 L1 coding region that suppress the HPV16 late 3'-splice site SA5639 in mitotic cells (Figure 1) [32,33,34]. There are multiple splicing silencer elements with AG-rich binding sites for hnRNP A1 in the HPV16 L1 coding region. One example is shown in Figure 2A [32]. In contrast, the first 17 nucleotides after SA5639 are required for splicing to SA5639 (Figure 2B), but the identity of proteins binding to this sequence is unknown [34]. Since high levels of hnRNP A1 are predicted to inhibit HPV16 L1 expression, one would expect high levels of hnRNP A1 in the lower layers of the cervical epithelium, and less in the superficial layers, which was also observed [42]. Furthermore, high-grade cervical lesions that contain persistent HPV16 infections that drive cell proliferation, but are non-productive, overexpress hnRNP A1, as do cervical cancer cells that are non-permissive for HPV16 production [42]. The splicing silencer elements in the HPV16 L1 coding region interacted specifically with an unidentified 65 kDa protein in addition to hnRNP A1 [34]. It has also been suggested that hnRNP A1 binds to a negative regulatory RNA element in the 3'-untranslated region of HPV16 late mRNAs [43]. In addition, hnRNP A1 also promotes splicing of early HPV16 mRNAs, specifically splicing between SD226 and SA409, whereas Sam68 promoted exon inclusion [44]. Thus, hnRNP A1 promotes HPV16 early mRNA splicing and inhibits HPV16 late mRNA splicing.

### 5.2. hnRNP A2/B1

Similarly to hnRNP A1, hnRNP A2/B1 binds to HPV16 splicing silencers that suppress HPV16 late mRNA splicing [31]. Together with proteins of the hnRNP D-family, hnRNP A2/B1 binds to splicing silencer elements upstream of HPV16 late 5'-splice site SD3632 [31], supporting the idea that hnRNP A2/B1 inhibits HPV16 late gene expression. However, overexpression of hnRNP A2/B1 may also induce HPV16 late gene expression [45], suggesting that hnRNP A2/B1 interferes with the function of hnRNP A1, or that posttranslational modifications of hnRNP A2/B1 determine its function. The effect of hnRNP A2/B1 on HPV16 RNA processing remains to be determined. 

### 5.3. hnRNP C1/C2, RALYL, and RALY

hnRNP C1 and C2 are produced from two alternatively spliced mRNAs expressed from the same gene and together with RALY and RALYL they belong to the hnRNP C family of hnRNPs [46]. hnRNP C1 and C2 have been shown to bind to an AU-rich RNA instability element in the UTR of the late mRNAs produced by the cutaneous HPV1 (Figure 2C) [47,48]. Binding was mapped to three penta-U nucleotides in the UTR (Figure 2C) [47,48] and overlapped with AUUUUA- binding sites for the HuR protein (see below). The results suggest that hnRNP C1/C2 control HPV1 late gene expression.

hnRNP C1 also binds directly to the U-rich region of the HPV16 early UTR *in vitro* and interacts with HPV16 early mRNAs in living cells (Figure 1 and Figure 2D) [49]. The HPV16 early UTR stimulates polyadenylation at pAE [26], but also appears to reduce the half-life of the early mRNAs [50]. Interestingly, overexpression of hnRNP C1 activates the suppressed HPV16 late 5'-splice site SD3632 in an HPV16 early UTR-dependent manner, suggesting that the HPV16 early UTR serves as a landing pad for cellular factors that control HPV16 late gene expression and alternative splicing [49]. Two other members of the hnRNP C family of proteins, RALY and RALYL, also activated HPV16 L1 mRNA splicing but to a lower extent [49]. Mechanistically, it was suggested that hnRNP C1 interferes with the splicing silencer complex that forms upstream of SD3632 to alleviate suppression [49]. Indeed, hnRNP C1 was able to interact with this splicing silencer complex that consisted of hnRNP D and hnRNP A2/B1 proteins [49]. hnRNP C1 therefore regulates HPV16 late gene expression by controlling HPV16 alternative splicing.

### 5.4. hnRNP D, hnRNP DL and hnRNP AB

hnRNP D, hnRNP DL and hnRNP AB all belong to the same hnRNP D family of hnRNPs [46]. The hnRNP D, hnRNP DL and hnRNP AB proteins were all shown to bind specifically to two AUAGUA-motifs present in a splicing silencer located immediately upstream of HPV16 late 5'-splice site SD3632 (Figure 1 and Figure 2E) [31]. In addition, hnRNP A2/B1 was present in this complex [31]. Interestingly, the hnRNP D proteins bound ATAGTA ssDNA and AUAGUA ssRNA with similar affinity and specificity [31]. The presence of the AUAGUA motifs was required for inhibition of SD3632, suggesting that the hnRNP D proteins that bind specifically to the AUAGUA motifs suppress SD3632 [31], thereby inhibiting production of HPV16 spliced L1 mRNAs. Furthermore, overexpression of hnRNP C1 appeared to interfere with this silencing complex and activated production of spliced L1 mRNA [49]. Collectively, the results are consistent with a role for hnRNP D proteins in the suppression and control of HPV16 late, L1 gene expression in non-terminally differentiated cells.

### 5.5. hnRNP E1/E2 (polyC Binding Proteins 1 and 2) and hnRNP K

hnRNP E1 and E2 have affinity for polyC and bind specifically to C-rich sequences in the HPV16 L2-coding region [51]. The same region bound hnRNP K, which also has affinity for C-rich sequences. Binding of hnRNP E1, E2 and hnRNP K to *in vitro* synthesized HPV16 L2 mRNAs inhibited translation of these mRNAs *in vitro* [51], but the role of these interactions in the control of HPV16 gene expression remains to be determined.

### 5.6. hnRNP H

hnRNP H binds specifically to triple-G motifs located downstream of the HPV16 early polyadenylation signal in the L2 coding region (Figure 1 and Figure 2F) [27,28]. This part of the L2 coding region stimulates polyadenylation at the early polyadenylation signal in HPV16 and in HPV31 [27,28,29,30]. The triple-G motifs enhanced polyadenylation at HPV16 pAE and insertion downstream of the pAE of a codon-modified L2 sequence that also destroyed the triple-G motifs resulted in an increased readthrough into the HPV16 late region at the pAE [27,28]. These results suggest that hnRNP H enhance polyadenylation of HPV16 early mRNAs, thereby inhibiting HPV16 late gene expression. Interestingly, the levels of hnRNP H in cervical epithelium drop in response to differentiation, and are elevated in high-grade cervical lesions and cervical cancer, thereby displaying an inverse correlation with HPV16 late gene expression [27,28]. hnRNP H therefore appears to be a suppressor of HPV16 late gene expression.

### 5.7. hnRNP I (Polypyrimidine Tract Binding Protein (PTB))

hnRNP I/PTB binds directly to the U-rich region in the HPV16 early UTR (Figure 2D) [26] and overexpression of hnRNP I/PTB causes a read-through into the HPV16 late region suggesting that hnRNP I/PTB interferes negatively with the HPV16 early polyadenylation signal pAE [52]. In addition, overexpression of hnRNP I/PTB also activated HPV16 late 5'-splice site SD3632 and induced splicing to late 3'-splice site SA5639 [52], but only under circumstances in which the splicing silencers at SA5639 had been mutationally inactivated, suggesting that PTB could relieve SD3632 inhibition, but could not activate late 3'-splice site SA53639. These results suggested that hnRNP I/PTB can control HPV16 late gene expression.

### 5.8. SRSF1 (ASF/SF2)

A strong splicing enhancer is located downstream of HPV16 3'-splice site SA3358 (Figure 1) [9,12,21,22]. This is one of the most efficiently used 3'-splice sites in the HPV16 genome despite its poor homology to a consensus 3'-splice site. Further experiments revealed that the corresponding sequences from HPV1, HPV4, HPV5, HPV6, HPV18 and HPV41, also displayed a strong splicing stimulatory effect when inserted downstream of HPV16 SA3358 [23]. Mutational inactivation of 10 predicted SRSF1 binding sites downstream of HPV16 3'-splice site SA3358 reduced splicing to SA3358, suggesting that SRSF1 interacted with the splicing enhancer [22]. Indeed, SRSF1 binds to the HPV16 splicing enhancer *in vitro* and in living cells [23]. Further mapping of the HPV16 enhancer activity revealed that one of the SRSF1 binding site accounted for the majority of the splicing enhancer activity (Figure 2G) [23]. Inactivation of this site resulted in lower production of HPV16 early mRNAs. If splicing silencers at the downstream, late 3'-splice site SA5639 were inactivated, mutational inactivation of the predicted SRSF1 binding sites at SA3358, resulted in redirection of splicing to SA5639 [23]. Overexpression of a mutant SRSF1 protein that binds RNA but lacks the RS-domain had a similar effect on HPV16 gene expression [22]. These results predict that SRSF1 would be overexpressed in HPV16 infected cells that produce primarily HPV16 early mRNAs. Indeed, it was observed that SRSF1 is overexpressed in HPV16-positive high-grade cervical lesions and in cervical cancer [42]. Furthermore, it was suggested that HPV16 E2 activates transcription of the SRSF1 gene [53]. It has also been reported that SRSF1 binds to the HPV16 late UTR, but this interaction appeared to be indirect and weak [54]. SRSF1 is also involved in the regulation of BPV-1 gene expression [55]. Taken together, published results suggest that SRSF1 is required for both HPV16 early and late gene expression.

### 5.9. SRSF3 (SRp20)

SRSF3 has been shown to bind to HPV16 sequences downstream of SA3358 (Figure 1) [25,56]. These sequences are AC-rich and are distinct from the SRSF1 binding site (Figure 2H). Interestingly, SRSF3 seems to inhibit splicing to HPV16 3'-splice site SA3358, either in its own right or by interfering with other SR proteins that enhance splicing to HPV16 SA3358 [25,56]. It is therefore pertinent to mention that SRSF3 is involved in the early to late switch in bovine papillomavirus (BPV) gene expression [25,56].

### 5.10. SRSF4 and SRSF6

Both SRSF4 and SRSF6 have been shown to bind sequences located downstream of HPV16 SA3358, but their roles in HPV16 splicing regulation remain to be determined [25,56].

### 5.11. SRSF9 (SRp30c)

SRSF9 binds to sequences downstream of HPV16 SA3358, but the exact binding site has not been determined (Figure 1) [24]. Overexpression of SRSF9 or a mutant SRSF9 protein with retained RNA binding activity but lacking the RS-domain, inhibited splicing to HPV16 SA3358 and redirected splicing to the downstream 3'-splice site SA5639 [24]. Since this redirection to late 3'-splice site SA5639 occurred efficiently even in the presence of multiple splicing silences in the L1 coding region, one may speculate that SRSF9 interferes with these splicing silencers to promote splicing to SA5639 [24]. SRFS9 may therefore both inhibit splicing to SA3358 and activate splicing to SA5639, thereby serving as an inducer of HPV16 late gene expression.

### 5.12. CstF64

CstF64 is a polyadenylation factor that binds to sequences downstream of cellular polyadenylation signals and stimulates polyadenylation. HPV16 and HPV31 both contain polyadenylation stimulatory RNA elements located in the L2 coding region downstream of the early polyadenyaltion signal (Figure 1) [27,28,29,30]. In both HPV16 and HPV31 these sequences interact with CstF64 [28,29], suggesting that CstF64 enhance polyadenylation of early HPV mRNAs and that the levels of CstF64 in the HPV infected cells may contribute to the control of mRNA read-through into the late region of the HPV16 and HPV31 genomes.

### 5.13. CUG-BP1

CUG-BP1 binds to a bipartite negative regulatory element in the HPV16 late untranslated region and acts synergistically with U1snRNP to mediate the inhibitory function of this RNA element (Figure 1) [36,38,39,57]. While U1snRNP binds to 5'-splice site like sequences in the element, CUG-BP1 binds to GU-rich sequences immediately downstream of the U1snRNP binding sites [36,38] (Figure 2I). A protein of the same as size as CUG-BP1 was shown to bind with sequence specificity to the late UTR elements of both HPV1 and HPV16 [40], suggesting that CUG-BP1 controls HPV late gene expression of both HPV1 and HPV16. It should be noted that the presence of inhibitory RNA elements in the late UTR appears to be a conserved property of HPVs, as is the presence of various permutations of the PuU_3-5_Pu motif, although the HPV16 late UTR had the greatest inhibitory activity when compared to other HPV types [40].

### 5.14. Fip1

Fip1 is a part of the cellular CPSF-polyadenylation factor and enhances polyadenylation by binding to U-rich sequences located upstream of cellular polyadenylation signals. The Fip1 protein binds directly to an exceptionally U-rich region in the HPV16 early UTR that has a stimulatory effect on HPV16 early polyadenylation (Figure 1 and Figure 2D) [26]. One may speculate that the enhancement of HPV16 early polyadenylation by the early UTR is mediated by the Fip1 protein.

### 5.15. HuR

The HuR protein binds AU-rich RNA instability elements on cellular and viral mRNAs. The UTR of late HPV1 mRNAs contains a classical AU-rich RNA instability element with UAUUUAU-motifs and penta-U nucleotides (Figure 2C) [47,58]. The HuR protein binds specifically to two UAUUUAU motifs and three UA/(C)UUUUUAU motifs in the HPV1 late UTR [59], but the functional significance of the binding is unknown. One may speculate that HuR stabilizes HPV1 late mRNAs or promotes their nuclear export. In support of this idea, the HPV1 AU-rich RNA element was less inhibitory in cell lines with high levels of HuR in the cytoplasm [60]. As mentioned above, hnRNP C1 also binds to the penta-U elements [47,48].

It has been suggested that HuR binds to the late 3'-UTR of HPV16 and HPV31 [61], possibly to GUUUG-motifs present in the HPV16 late UTR as the typical HuR binding sites UAUUUAU are absent from the HPV16 late UTR (Figure 2I). However, other investigators failed to detect HuR binding of to the HPV16 late UTR [36,40]. Overexpression of HuR can induce HPV16 late gene expression [62]. This effect has also been observed in the absence of HPV16 late UTR sequences [19], suggesting that HuR controls HPV16 late gene expression through other HPV16 sequences as well.

### 5.16. PABP

PolyA binding protein (PABP) is required for elongation of the polyA tail at the 3'-end of cellular mRNAs and for efficient mRNA translation. We have shown that the AU-rich RNA element in the HPV1 late UTR inhibits translation in addition to its RNA-destabilizing effect (Figure 2C) [63]. The AU-rich RNA element also interacts with PABP [63], indicating that the HPV1 late UTR may interfere with the translation stimulatory function of PABP.

### 5.17. U1snRNP

In addition to the U-rich region that binds CUG-BP, the bipartite negative RNA element in the HPV16 late UTR element encodes 5'-splice site-like sequences [38,39,57]. The inhibitory function of the HPV16 late UTR depends on both the 5'-splice like sequences and the U-rich region that binds CUG-BP. Also the bovine papillomavirus type 1 late UTR encodes an unutilized 5'-splice element [38]. The interaction of the 5'-splice site like sequences with U1snRNP inhibits polyadenylation at the late polyA signal through an interaction between the U1-70K protein of U1snRNP and the polyA polymerase [37]. U1snRNP is therefore a suppressor of HPV16 late gene expression.

### 5.18. U2AF65

The splicing factor U2AF65 has been reported to bind GU-rich sequences immediately downstream of the U1snRNP binding sites in the HPV16 late UTR [54,62], but this interaction was not detected by other investigators [36,40]. However, increased levels of U2AF65 are observed in HPV-positive, high-grade cervical lesions [42], which is consistent with an inhibitory effect on HPV16 late gene expression. Furthermore, U2AF65 binds to a splicing silencer in BPV1 and inhibits splicing of BPV1 early and late mRNAs [64]. It is unclear if U2AF65 contributes to control of HPV16 late gene expression.

## 6. Interactions of the HPV1 E4 Protein with SR-Protein Kinase SRPK1

HPV E4 proteins colocalize with the cellular SR-proteins kinase 1 (SRPK1) in human cells [65]. Since SRPK1 phosphorylates splicing factors of SR-protein family, HPV E4 may contribute to HPV splicing regulation. Indeed, HPV1 E4 has been shown to bind to SRPK1 and inhibit the phosphorylation *in vitro* of SR proteins and the HPV E2 protein by SRPK1 [66]. However, direct effects of E4 on HPV mRNA processing have not been reported.

In this regard, it is of interest to compare with the adenovirus E4orf4 protein binds to SR proteins and to the major cellular phosphatase PP2A, which results in dephosphorylation of SR proteins [67]. As a consequence, adenovirus RNA processing is altered and adenovirus late gene expression is activated [67]. Intriguingly, overexpression of adenovirus E4orf4 also altered HPV16 RNA splicing and caused an induction of HPV16 late L1 mRNA splicing [68]. Therefore, dephosphorylation of SR proteins induces HPV16 late gene expression. Interestingly, mucosal epithelial cell differentiation results in a decrease in the levels of phosphorylated SR proteins, while progression to cervical cancer via cervical lesions of varying grades results in a progressively higher SR protein levels [42,56,69]. It is reasonable to speculate that inactivation or down regulation of certain SR proteins, causes induction of HPV late gene expression.

## 7. The HPV E2 Protein—An RNA binding, RNA Processing Factor?

The HPV E2 proteins is a DNA binding protein that binds to HPV LCR DNA to control transcription, and to recruit the cellular DNA polymerase together with HPV E1 to replicate the HPV genomic DNA [70]. It also binds cellular chromatin to control partitioning of the HPV DNA genome during cell division [70]. In addition, accumulating data support the idea that E2 is an RNA processing factor that may control HPV- and cellular-RNA splicing [18] and HPV early polyadenylation [19]. HPV E2 may carry out many of its function through protein-protein interactions but E2 may also interact with RNA directly. HPV E2 interacts with multiple splicing factors (SR proteins) [71] and binds RNA *in vitro* and inhibits *in vitro* splicing [18]. A recent report indicates that overexpression of E2 in a human cancer cell line altered splicing in these cells [72], but it remains to be determined if this was through protein-protein or protein-RNA interactions. Although in theory, the results could be explained by the observation that E2 increases expression of SRSF1 gene [53]. In addition to potential effects of E2 on splicing, it has been shown that E2 inhibits HPV16 early polyadenylation thereby causing read-through into the late region of the HPV16 genome and activation of HPV16 late gene expression [19]. E2 affected the conformation of the polyadenylation complex *in vitro*, suggesting that E2 interferes with cellular polyadenylation factors at the HPV16 early polyA signal pAE, thereby activating HPV late gene expression [19]. The expression levels of E2 may therefore control the switch from HPV16 early to late gene expression [19]. The recently described differentiation-dependent increase in the levels of E2 in HPV-infected cervical epithelium [73] is consistent with the idea that E2 contributes to induction of HPV late gene expression.

## 8. Do Epigenetic Properties of the HPV Genome Contribute to the Control of HPV RNA Splicing and Polyadenylation?

Epigenetic changes of the HPV16 genome have been described and include: CpG methylation of HPV DNA, and posttranslational modifications of HPV16-associated histones. More than hundred CpG motifs that could potentially be methylated are present in non-coding as well as protein-coding regions of the HPV16 genome, and they are unevenly distributed over the HPV16 genome [74]. Although methylation of these sites change over time in an infected individual and methylation of CpG sites in the E2, L1 and L2 coding regions appear to increase as high-grade lesions develop, the significance of HPV DNA methylation remains obscure [74]. DNA methylation could either prevent proteins from binding HPV DNA, or attract proteins that recognize methylated DNA. Such factors could potentially interact with cellular splicing and/or polyadenylation factors and contribute to the control of HPV RNA processing. However, DNA methyl transferase inhibitors did not affect HPV16 late gene expression in HPV16 transfected cervical cancer cells [75], but further experiments are needed to role out HPV DNA methylation in HPV RNA processing. Interestingly, a recent publication on an HPV18 deletion mutant lacking a CCCTC-binding factor (CTCF) DNA binding site in the E2 coding region described effects on HPV18 early mRNA splicing [76]. Although one cannot exclude that the mutations affected splicing regulatory elements, the results suggest that an HPV18 DNA binding protein could be involved in HPV18 RNA processing [76]. Finally, modifications of HPV16 DNA bound histones may also contribute to HPV16 RNA processing by recruiting splicing factors to strategic positions on the HPV16 genome that codes for splices sites, polyadenylation signals or regulatory RNA elements in order to facilitate interactions between RNA binding proteins and de novo synthesized mRNAs [75]. Indeed, it has been shown that some histone marks are unevenly distributed over the HPV16 [75] and HPV18 [77] genomes and that activation of HPV16 gene expression by HDAC inhibitors altered acetylation of HPV16 intragenic histones [75]. It remains to be determined if epigenetic marks on HPV16 contribute to regulation of HPV16 mRNA processing.

## 9. Conclusions

Cellular RNA-binding proteins control HPV16 gene regulation at the level of RNA processing by binding to the HPV16 mRNAs. In this capacity, cellular RNA binding proteins play an important role in the natural course of the HPV16 infection, as well as during progression to cancer. Indeed, expression levels of many RNA binding proteins are altered in both premalignant lesions caused by HPV16 as well as in cervical cancer. A better understanding of the control of HPV16 gene expression by RNA binding proteins is necessary in order to understand how HPV16 causes disease. This research may uncover novel targets for antiviral drugs to HPV16 as well as predictive biomarkers for women at risk of developing cervical cancer.
